# Mild traumatic brain injury, PTSD symptom severity, and behavioral dyscontrol: a LIMBIC-CENC study

**DOI:** 10.3389/fneur.2023.1286961

**Published:** 2024-01-11

**Authors:** Kelsee M. Stromberg, Sarah L. Martindale, William C. Walker, Zhining Ou, Terri K. Pogoda, Shannon R. Miles, Clara E. Dismuke-Greer, Kathleen F. Carlson, Jared A. Rowland, Maya E. O’Neil, Mary Jo Pugh

**Affiliations:** ^1^Informatics, Decision-Enhancement, and Analytic Sciences (IDEAS) Center, VA Salt Lake City Health Care System, Salt Lake City, UT, United States; ^2^Department of Internal Medicine, Division of Epidemiology, Spencer Fox Eccles School of Medicine, University of Utah, Salt Lake City, UT, United States; ^3^Research and Academic Affairs Service Line, W. G. (Bill) Hefner VA Healthcare System, Salisbury, NC, United States; ^4^Veterans Integrated Service Networks (VISN)-6 Mid-Atlantic Mental Illness, Research Education and Clinical Center (MIRECC), Durham, NC, United States; ^5^Department of Physiology and Pharmacology, Wake Forest School of Medicine, Winston-Salem, NC, United States; ^6^Department of Physical Medicine and Rehabilitation (PM&R), School of Medicine, Virginia Commonwealth University, Richmond, VA, United States; ^7^Department of PM&R, Central Virginia VA Health Care System, Richmond, VA, United States; ^8^Center for Healthcare Organization and Implementation Research, VA Boston Healthcare System, Boston, MA, United States; ^9^Department of Health Law, Policy and Management, Boston University School of Public Health, Boston, MA, United States; ^10^Mental Health and Behavioral Sciences Services, James A. Haley Veterans’ Hospital, Tampa, FL, United States; ^11^Department of Psychiatry and Behavioral Neurosciences, Morsani College of Medicine, University of South Florida, Tampa, FL, United States; ^12^Health Economics Resource Center (HERC), Ci2i, VA Palo Alto Health Care System, Menlo Park, CA, United States; ^13^VA HSR&D Center to Improve Veteran Involvement in Care (CIVIC) and RR&D National Center for Rehabilitative Auditory Research (NCRAR), Veterans Affairs Portland Health Care System, Portland, OR, United States; ^14^Oregon Health and Science University – Portland State University School of Public Health, Oregon Health & Science University, Portland, OR, United States; ^15^Department of Neurobiology and Anatomy, Wake Forest School of Medicine, Winston-Salem, NC, United States; ^16^Department of Psychiatry, Oregon Health & Science University, Portland, OR, United States; ^17^Medical Informatics and Clinical Epidemiology, Oregon Health & Science University, Portland, OR, United States

**Keywords:** dysregulation, concussion, military members, transition introduction, TBI – traumatic brain injury

## Abstract

**Background:**

Behavioral dyscontrol occurs commonly in the general population and in United States service members and Veterans (SM/V). This condition merits special attention in SM/V, particularly in the aftermath of deployments. Military deployments frequently give rise to posttraumatic stress disorder (PTSD) and deployment-related mild TBI traumatic brain injury (TBI), potentially leading to manifestations of behavioral dyscontrol.

**Objective:**

Examine associations among PTSD symptom severity, deployment-related mild traumatic brain injury, and behavioral dyscontrol among SM/V.

**Design:**

Secondary cross-sectional data analysis from the Long-Term Impact of Military-Relevant Brain Injury Consortium – Chronic Effects of Neurotrauma Consortium prospective longitudinal study among SM/V (*N* = 1,808).

**Methods:**

Univariable and multivariable linear regression models assessed the association and interaction effects between PTSD symptom severity, as assessed by the PTSD Checklist for the Diagnostic and Statistical Manual, 5th edition (PCL-5), and deployment-related mild TBI on behavioral dyscontrol, adjusting for demographics, pain, social support, resilience, and general self-efficacy.

**Results:**

Among the 1,808 individuals in our sample, PTSD symptom severity (*B* = 0.23, 95% CI: 0.22, 0.25, *p* < 0.001) and deployment-related mild TBI (*B* = 3.27, 95% CI: 2.63, 3.90, *p* < 0.001) were significantly associated with behavioral dyscontrol in univariable analysis. Interaction effects were significant between PTSD symptom severity and deployment mild TBI (*B* = −0.03, 95% CI: −0.06, −0.01, *p* = 0.029) in multivariable analysis, indicating that the effect of mild TBI on behavioral dyscontrol is no longer significant among those with a PCL-5 score > 22.96.

**Conclusion:**

Results indicated an association between PTSD symptom severity, deployment-related mild TBI, and behavioral dyscontrol among SM/V. Notably, the effect of deployment-related mild TBI was pronounced for individuals with lower PTSD symptom severity. Higher social support scores were associated with lower dyscontrol, emphasizing the potential for social support to be a protective factor. General self-efficacy was also associated with reduced behavioral dyscontrol.

## Introduction

Behavioral dyscontrol is a challenging clinical problem that cuts across traditional diagnostic boundaries. Wotzel and Arciniegas provide an overview of the literature that encompasses a range of terms describing behavioral dyscontrol following injury, which include emotional lability, irritability, anger, aggression, and challenges in self-regulation (1). These various symptoms denoting behavioral dyscontrol present clinically challenging sequelae that frequently impede rehabilitation efforts, disrupt social support networks and compromise optimal recovery (1, 2). The absence of a standardized or universally accepted definition and the limitations in delineating mental and behavioral presentations following TBI contribute to the complexity of behavioral dyscontrol (1). Although a multitude of factors can influence the development and severity of behavioral dyscontrol, posttraumatic stress disorder (PTSD) and traumatic brain injury (TBI) have been identified as important contributors (2, 3). Veterans who have served in combat deployments are at risk of experiencing TBI and developing PTSD, which also increases their risk for behavioral dyscontrol (4). Understanding potential links among PTSD, deployment mild TBI, and behavioral dyscontrol can inform post-deployment healthcare delivery for Service members and Veterans (SM/Vs).

Deployment-related mild TBI can disrupt neural pathways crucial for impulse control (2). Due to heterogeneity in injury, mild TBI may manifest in various behavioral dyscontrol symptoms, including explosive outbursts, verbal and physical aggression, impaired judgment and planning abilities, and limited self-awareness (1, 2, 5). Similarly, some Veterans with PTSD report experiencing challenges with self-regulation, which may also be associated with symptoms of behavioral dyscontrol such as agitation and explosive behavior (1, 2, 5).

The present analysis aimed to investigate the associations between history of deployment-related mild TBI, PTSD symptoms, and behavioral dyscontrol in a cohort of combat SM/Vs. Given that prior work identified poorer outcomes for SM/Vs with TBI and high PTSD symptom severity, we hypothesized that behavioral dyscontrol would be highest among those with deployment-related mild TBI and high PTSD symptom severity (6–8).

## Methods

### Design

This cross-sectional study utilized data from the baseline visit of the longitudinal, multi-center Prospective Longitudinal Study (PLS) conducted by the Long-term Impact of Military-relevant Brain Injury Consortium-Chronic Effects of Neurotrauma Consortium (LIMBIC-CENC) (9). The primary objective of the PLS is to assess the effects of mild TBI (s) and other comorbidities on neurological and psychological outcomes among combat-exposed SM/Vs (10). During the baseline visit, participants completed a comprehensive assessment, which included structured interviews, questionnaires, neuropsychological testing, and biometric measurements (11).

### Participants

The PLS is conducted across 11 recruitment sites. Enrollment is ongoing, with over 2,000 SM/Vs enrolled. Participants were recruited primarily through targeted mailings. Eligible individuals included SM/Vs who had deployed to a combat zone, were at least 18 years of age, and had no history of moderate to severe TBI or major neurological or psychiatric illnesses resulting in a significant long-term decrease in functional status (e.g., schizophrenia, spinal cord injury) (9). Common comorbidities, including PTSD and depression, were permitted. The study obtained approval from the regional Institutional Review Boards of the participating facilities, and written consent was obtained from all participants before any procedures were conducted. The available sample size for the presented analyses at the time of database extraction was *N* = 2,069. Participants were excluded from these analyses for missing data on key measures (*n* = 118) and for noncredible symptom-reporting profiles on the mild Brain Injury Atypical Symptoms (mBIAS) questionnaire (10) (*n* = 143). These exclusions left a final analytic sample size of *N* = 1,808. Within this cohort, 278 experienced TBI during deployment only, 680 TBI from both deployment and non-deployment settings, 507 had TBI solely in non-deployment settings, and 343 individuals had no history of TBI.

### Measures

The primary outcome, behavioral dyscontrol, was assessed by self-report using the 10-item Traumatic Brain Injury Quality of Life (TBIQOL) questionnaire, which measures emotional and behavioral dyscontrol, including disinhibition, emotional lability, irritability, impatience, and impulsiveness (11, 12)—the primary characteristics of behavioral dyscontrol. The total score ranges from 10 to 50, with higher scores indicating higher levels of dyscontrol (12).

### Independent variables

History of deployment-related mild TBI was evaluated using the Virginia Commonwealth University Retrospective Concussion Diagnostic Interview (VCU-rCDI)—a structured interview developed for the PLS to facilitate the classification of all potential concussive events (PCEs) experienced throughout an individual’s lifetime (13). PCEs are first identified via a modified OSU TBI ID interview version (13). Each PCE is evaluated with the VCU-rCDI to determine if it meets the criteria for mild TBI and to gather information on setting, mechanism, and other clinical characteristics (13). Diagnostic determinations were consistent with the VA/ Department of Defense (DoD) standard definition of mild TBI and the American Congress of Rehabilitation Medicine guidelines (14, 15). Variables utilized for this analysis included deployment and non-deployment designation for each mild TBI based on whether the TBI occurred during a combat deployment or some other time during life.

PTSD symptom severity was assessed using the PTSD Checklist for the Diagnostic and Statistical Manual, 5th edition (PCL-5) (16). This 20-item self-report measure utilizes a 5-point Likert scale to evaluate how bothered an individual has been by symptoms associated with PTSD in the past 30 days. Total scores range from 0 to 80, with greater scores indicating greater symptom severity (16). Participants scoring 33 or higher on the PCL-5 were classified as positive for probable PTSD (16).

### Covariates

Previous studies have identified key symptoms associated with PTSD, mild TBI, and behavioral dyscontrol (17, 18) that were chosen as covariates for the present analysis.

The Euroqol 5 measures pain/discomfort on a 5-point ordinal scale, allowing respondents to assess their current pain as none, slight, moderate, severe, or extreme (19). Meanwhile, the TBI Qol Pain Interference Short-Form comprises a 10-item questionnaire prompting participants to rate the extent of pain interference across functions like family life, daily tasks, mental health, and overall quality of life. Responses are noted on a 5-point ordinal scale (1–5), and total scores range from 10 to 50 points, with higher scores reflecting greater interference (19).

General Self-Efficacy (GSE) was measured using a 10-item self-reported questionnaire that measures an individual’s perceived ability to solve problems and achieve their goals (20). Response options range from 1 (Not at all true) to 5 (Exactly true), with a total score range of 10–50 (20).

The Deployment Risk and Resilience Inventory-2 (DRRI-2) Social Support subscale is a comprehensive self-report assessment tool designed to evaluate the degree of social support available to individuals, particularly those who have experienced military deployment and related challenges (21). It encompasses a range of factors that contribute to an individual’s perception of social support, including emotional, instrumental, and informational assistance from family members, friends, and peers (21). The subscale aims to quantify the extent to which individuals perceive themselves as having access to a robust support network during and after deployment. Respondents are asked to rate their level of agreement or frequency on a Likert-type scale from 1 to 5, where higher scores indicate higher levels of perceived social support (21).

Lifetime Mild TBI history is assessed in LIMBIC-CENC PLS using a validated process, cataloging each participant’s potential concussive events through a modified Ohio State University TBI Identification (OSU TBI-ID) (12). A retrospective Concussion Diagnostic Interview generates a preliminary algorithm based TBI diagnosis, which undergoes rigorous review against medical records by a centralized expert committee. Final determinations align with the VA/DoD common definition of mild TBI.

Blast mechanisms are classified as blast-related or not, and if blast-related, as pure blast or mixed blast-blunt. Subclassifications for analysis include 1–2 mild TBIs vs. 3+ (repetitive mild TBI), mild TBIs with or without PTA, blast-related or not, and pure blast vs. non-blast/mixed blast-blunt mild TBIs (12).

We also included sociodemographic factors (age, sex, education, and race/ethnicity) selected based on the existing literature to adjust for their potential impact in multivariable analysis.

### Statistical methods

Demographic and clinical outcomes are reported using means and standard deviation (SD), medians and interquartile ranges (IQR), ranges for continuous variables, and counts and percentages for categorical variables. To compare these variables between deployment-related mild TBI exposure (yes/no) group, we used non-parametric Wilcoxon rank sum tests for continuous variables and chi-square or Fisher’s exact tests for categorical variables, as appropriate. There were no independent or interaction effects of non-deployment-related mild TBI on behavioral dyscontrol; therefore, non-deployment TBI was excluded from the analysis. Univariable and multivariable linear regression models assessed independent and interactive associations between variables of interest and behavioral dyscontrol. The multivariable model included the interaction term and adjusted for age, sex, ethnicity, race, Euroqol 5 – pain dimension scores, DRRI-2 Social Support scores, and GSE scores. Multicollinearity was considered tolerable if the generalized variance inflation factor (GVIF) was <2.24 (22). Johnson-Neyman analysis probed significant interaction effects. We report Betas (B) with 95% confidence intervals (CI) for each analysis. Statistical significance was assessed at the 0.05 level, and all analyses were performed using R v. 4.1.2 (23).

## Results

In our analytic sample of 1,808 individuals, 53% (*n* = 958) had deployment-related mild TBI, and 47% (*n* = 850) had no history of deployment mild TBI. Studying both deployment- and non-deployment-related mild TBI is fundamental for a comprehensive understanding of the diverse causes, mechanisms, and long-term impacts of TBI and the individuals affected by these injuries (24, 25). Table 1 presents the demographic characteristics of the sample, stratified by deployment-related mild TBI status. Most participants were male (87.0%) and White (73.0%), with a median age of 39 years (IQR: 33, 48). There were significant differences in behavioral dyscontrol scores between SM/Vs with (*M* = 23.6, SD = 6.8) versus without (*M* = 20.4, SD = 6.9) history of deployment-related mild TBI. Social support scores were higher among SM/Vs without (*M* = 40.1, SD = 7.9) compared to those with a history of deployment-related mild TBI (*M* = 38.1, SD = 8.1). Finally, SM/Vs without a history of deployment-related mild TBI reported greater self-efficacy (*M* = 32.7, SD = 4.7) than SM/Vs with a history of deployment-related mild TBI (*M* = 31.4, SD = 4.8).

**Table 1:** Summary of demographic variables stratified by deployment-related mild TBI.

Variable	All (*N* = 1808)	No deployment-related mild TBI: *N* = 850	Deployment-related mild TBI: *N* = 958	*p*-value
Age at baseline (yr)				
Mean (SD)	41.2 (10.0)	42.1 (10.4)	40.3 (9.5)	<0.001^k^
Median (IQR)	39.0 (33.0, 48.0)	41.0 (34.0, 50.0)	39.0 (33.0, 47.0)	–
Range	(22.0, 76.0)	(23.0, 76.0)	(22.0, 72.0)	–
Ethnicity				
Hispanic or Latino	299 (17%)	122 (14.4%)	177 (18.5%)	0.014^c^
Not Hispanic or Latino	1486 (82%)	721 (84.8%)	765 (79.9%)	–
Not Hispanic or Latino	23 (1%)	7 (0.8%)	16 (1.7%)	–
Race				
White	1314 (73%)	618 (72.7%)	696 (72.7%)	0.052^c^
Black/African American	328 (18%)	159 (18.7%)	169 (17.6%)	–
Asian/American Indian/Alaska Native/ or Pacific Islander	59 (3%)	34 (4%)	25 (2.6%)	–
Don’t know/Not sure/Refused/Other	107 (6%)	39 (4.6%)	68 (7.1%)	–
Gender				
Female	233 (13%)	156 (18.4%)	77 (8%)	0.001^c^
male	1574 (87%)	694 (81.6%)	880 (91.9%)	–
Marital status				
Never married	264 (15%)	134 (15.8%)	130 (13.6%)	0.005^s^
A member of an unmarried couple	25 (1%)	17 (2%)	8 (0.8%)	–
Married	1101 (61%)	514 (60.5%)	587 (61.3%)	–
Divorced	326 (18%)	150 (17.6%)	176 (18.4%)	–
Separated	75 (4%)	25 (2.9%)	50 (5.2%)	–
Widowed	14 (1%)	10 (1.2%)	4 (0.4%)	–
Refused	3 (0%)	0 (0%)	3 (0.3%)	–
Emotional and behavioral dyscontrol total score				
Mean (SD)	22.1 (7.0)	20.4 (6.9)	23.6 (6.8)	<0.001^k^
Median (IQR)	22.0 (17.0, 27.0)	20.0 (15.0, 25.0)	23.0 (18.0, 28.0)	–
Range	(10.0, 45.0)	(10.0, 42.0)	(10.0, 45.0)	–
Pain / Discomfort dimension				
Mean (SD)	2.4 (0.9)	2.2 (0.9)	2.6 (0.9)	<0.001^k^
Median (IQR)	2.0 (2.0, 3.0)	2.0 (1.0, 3.0)	2.0 (2.0, 3.0)	–
Range	(1.0, 5.0)	(1.0, 5.0)	(1.0, 5.0)	–
Social support				
Mean (SD)	39.1 (8.1)	40.1 (7.9)	38.1 (8.1)	<0.001^k^
Median (IQR)	40.0 (34.0, 45.0)	41.0 (36.0, 46.0)	39.0 (33.0, 44.0)	–
Range	(10.0, 50.0)	(10.0, 50.0)	(10.0, 50.0)	–
General self-efficacy				
Mean (SD)	32.0 (4.8)	32.7 (4.7)	31.4 (4.8)	<0.001^k^
Median (IQR)	32.0 (29.0, 36.0)	32.0 (30.0, 37.0)	31.0 (29.0, 35.0)	–
Range	(12.0, 40.0)	(12.0, 40.0)	(14.0, 40.0)	–
PTSD				
No	1214 (67%)	669 (78.7%)	545 (56.9%)	<0.001^c^
Yes	594 (33%)	181 (21.3%)	413 (43.1%)	–
PTSD symptom severity score				
Total PTSD symptom severity score (out of 80 pts) PCL5_TOT: Mean (SD)	25.1 (18.4)	18.9 (16.9)	30.6 (18.0)	<0.001^k^
Median (IQR)	22.0 (10.0, 38.0)	14.0 (5.0, 29.0)	29.0 (16.0, 44.0)	–
Range	(0.0, 78.0)	(0.0, 76.0)	(0.0, 78.0)	–

Number of missing values in the No Deployment-related mild TBI/Deployment-related mild TBI groups: Gender: = n = 0/n = 1, Pain / discomfort dimension: n = 0/n = 1, PTSD symptom severity score (out of 80 pts): n =10/n = 6. ^c^ Chi-squared test, ^k^ Kruskal-Wallis test, ^f^ Fisher’s exact test, ^s^ Chi-squared test by Montecarlo simulation.

**Figure 1:**
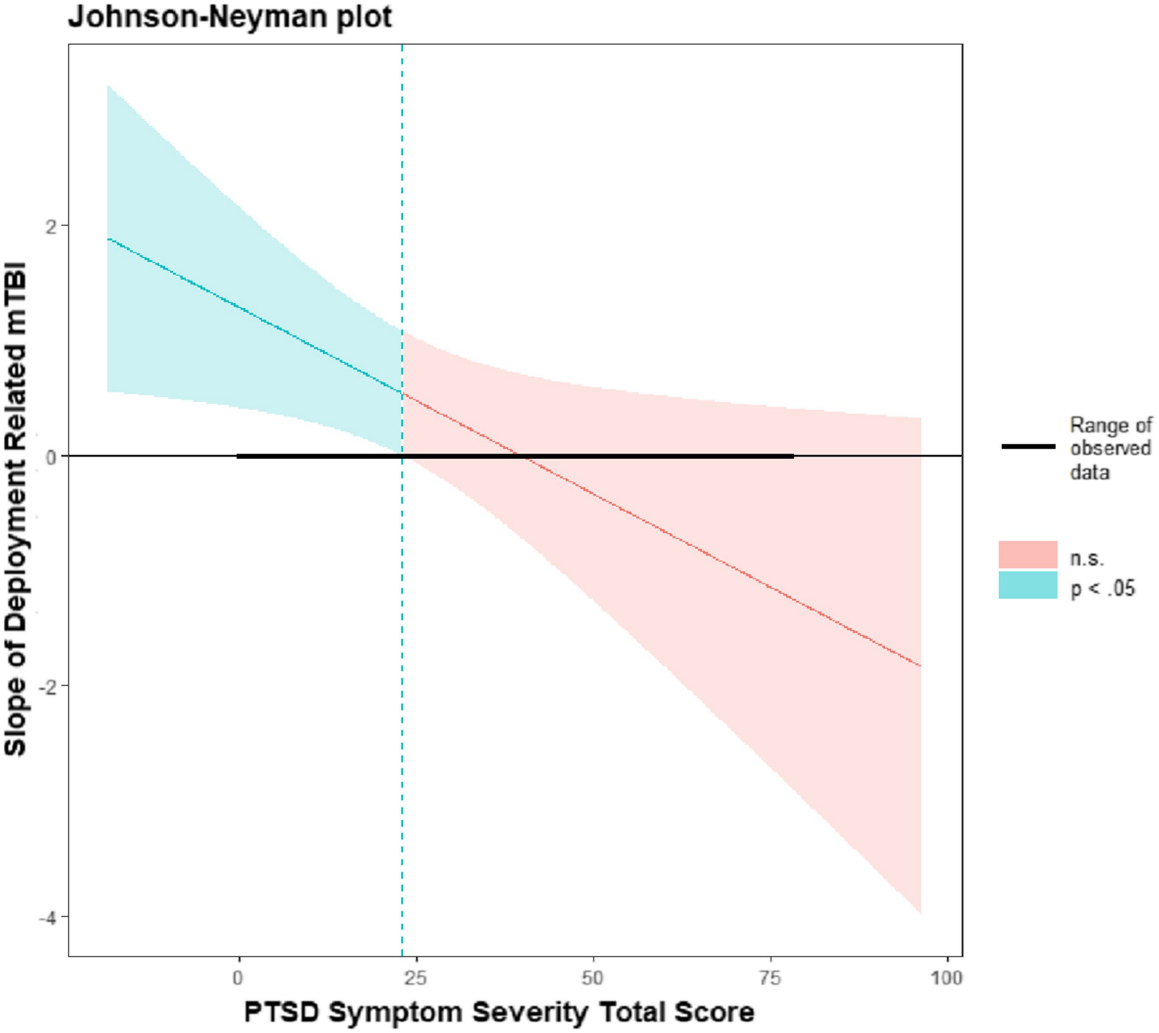
Johnson-Neyman intervals.

Table 2 presents linear regression models among SM/Vs with deployment-related mild TBI. In the univariable analyses, the presence of deployment-related mild TBI (*B* = 3.27, *p* < 0.001 CI [2.63, 3.90]) and PTSD symptom severity (Beta = 0.23, 95% CI: 0.22, 0.25, *p* < 0.001) were each independently associated with behavioral dyscontrol. Multivariable analysis indicated a significant interaction effect (*B* = −0.03, 95% CI: −0.06, −0.01, *p* < 0.001) between deployment-related mild TBI and PTSD symptom severity. Johnson-Neyman analysis (see Figure 1) indicated a critical score of 22.96, such that the interaction effect was significant when PCL-5 scores were between 0 and 22.96. This indicates that the impact of deployment-related mild TBI was significantly associated with behavioral dyscontrol when PCL-5 scores were ≤ 22.96.

**Table 2:** Behavioral dyscontrol score by deployment-related mild and PTSD.

Variable	Univariable coefficient (95% CI)	*p*-value	*N* used	Multivariable coefficient (95% CI)	*p*-value
Deployment-related TBI	3.27 (2.63, 3.90)	<0.001	1808	1.38 (0.36, 2.41)	0.008
PTSD	0.23 (0.22, 0.25)	<0.001	1800	0.21 (0.18, 0.23)	<0.001
Deployment-related TBI × PTSD	–	–	–	−0.03 (−0.06, 0.00)	0.046
Age at baseline (yr)	−0.06 (−0.09, −0.03)	<0.001	1808	−0.02 (−0.06, 0.02)	0.34
Pain/Discomfort dimension	2.45 (2.11, 2.79)	<0.001	1807	0.26 (−0.06, 0.57)	0.11
Social Support	−0.29 (−0.33, −0.26)	<0.001	1808	−0.06 (−0.09, −0.02)	<0.001
Ethnicity					
Not Hispanic or Latino	−0.68 (−1.55, 0.19)	0.13	1808	0.60 (−0.12, 1.31)	0.10
Do not know/Not sure/Refused	−1.43 (−4.41, 1.55)	0.35	1808	−0.35 (−2.69, 2.00)	0.77
Hispanic	Reference			Reference	
Race					
Asian/Pacific Islander/American Indian or Alaska Native	−0.61 (−2.45, 1.22)	0.51	1808	0.15 (−1.26, 1.55)	0.84
Black or African American	−0.48 (−1.33, 0.37)	0.27	1808	−1.79 (−2.46, −1.13)	<0.001
Do not know/Not sure/Refused/Other	0.17 (−1.22, 1.55)	0.81	1808	−0.35 (−1.53, 0.83)	0.56
White	Reference			Reference	
Gender: Male	0.19 (−0.78, 1.15)	0.71	1807	0.25 (−0.52, 1.02)	0.52
General Self-Efficacy	−0.64 (−0.70, −0.58)	<0.001	1808	−0.24 (−0.30, −0.17)	<0.001
Number of mild TBI^a^	0.64 (0.47, 0.80)	<0.001	1808	–	–
Blast-related TBI					
No TBI at all	−3.69 (−4.95, −2.43)	<0.001	1808	0.80 (−0.43, 2.03)	0.20
Non-Blast only	−1.46 (−2.60, −0.33)	0.012	1808	1.50 (0.51, 2.50)	0.003
Blast and Non-blast	0.22 (−0.98, 1.42)	0.72	1808	1.04 (0.09, 1.98)	0.031
Blast only	Reference			Reference	
Years of service	−0.06 (−0.10, −0.03)	<0.001	1807	0.00 (−0.0.4, 0.04)	0.98
Mental health treatment in the past 6 months					
- No response/Don’t know/Not sure	−2.96 (−10.58, 4.66)	0.45	1808	−1.00 (−8.47, 6.47)	0.79
Yes	4.09 (3.47, 4.71)	<0.001	1808	−0.06 (−0.63, 0.51)	0.83
No	Reference			Reference	

^a^ The number of mild TBI is the same as number of lifetime TBI if none of the TBIs are moderate-severe.

Among SM/Vs who experienced both blast and non-blast deployment-related mild TBI, but no PTSD, dyscontrol scores were found to be 1.04 points higher compared to those with blast-only deployment-related mild TBI (*B* = 1.04, 95% CI: 0.09, 1.98, *p* = 0.005).

Dyscontrol scores among SM/Vs with deployment-related mild TBI but no PTSD symptoms, were 1.38 points higher than those with deployment-related mild TBI after adjusting for other covariates (*B* = 1.38, 95% CI: 0.36, 2.41, *p* = 0.005). Among SM/Vs without deployment-related TBI (reference group), each one-point increase in the PTSD total score was associated with a 0.21-point increase in dyscontrol scores (*B* = 0.21, 95% CI: 0.18, 0.23, *p* < 0.001). Furthermore, accounting for other variables, there was a decrease of 0.24 points in dyscontrol scores (*B* = −0.24, 95% CI: −0.30, −0.17, *p* < 0.001) for every one-point increase in general self-efficacy scores. Similar findings were obtained for social support scores (*B* = −0.06, 95% CI: −0.09, −0.02, *p* < 0.001).

**Figure 2:**
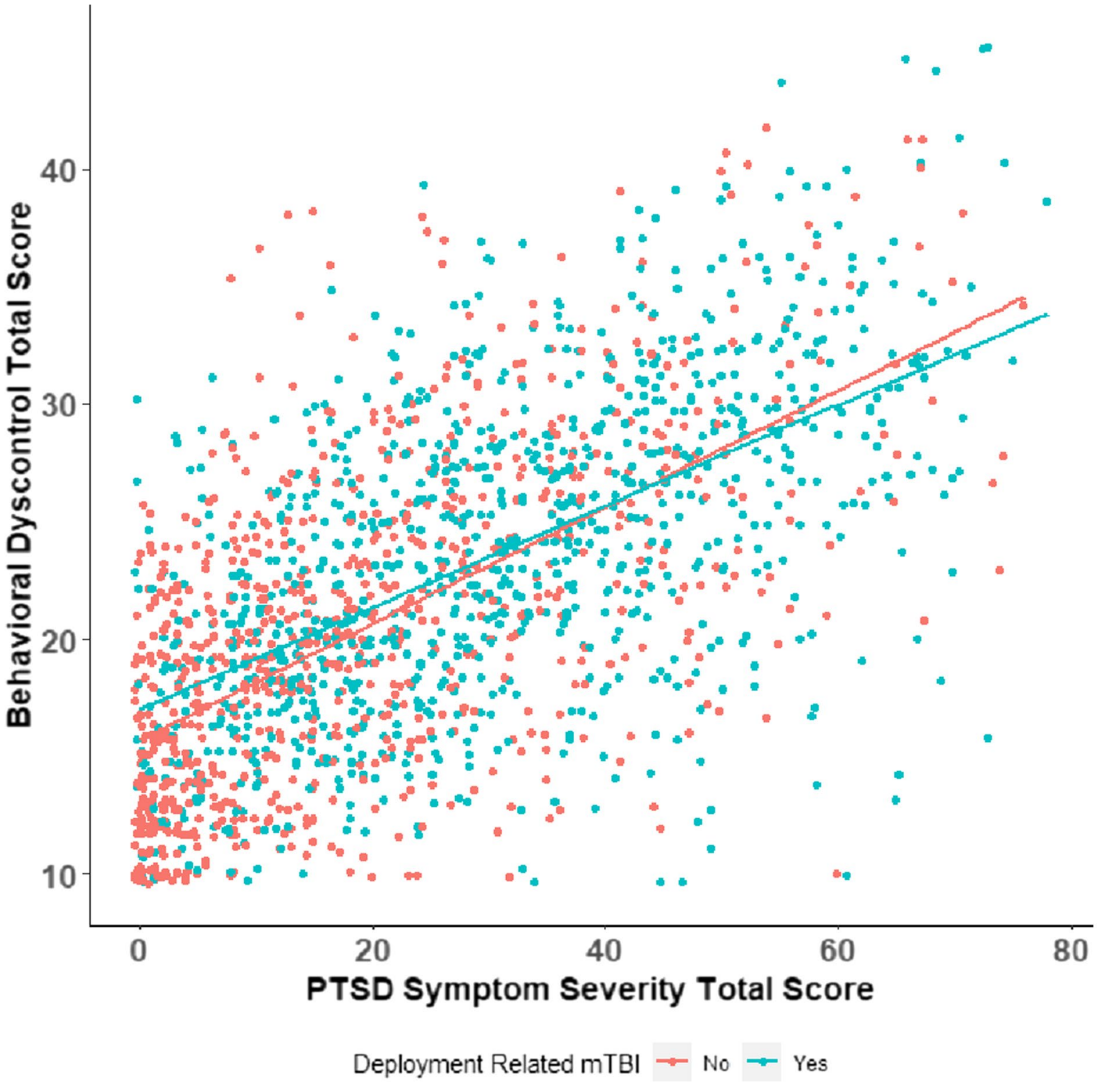
Interaction plot of deployment mild TBI and PTSD symptom severity.

Figure 2 interaction effect between deployment-related mild TBI status and PTSD symptom severity scores on behavioral dyscontrol. The slope of PTSD severity scores is steeper in SM/Vs without deployment-related mild TBI than those with no deployment-related mild TBI have lower observed behavioral dyscontrol total scores.

## Discussion

We hypothesized that SM/Vs with greater PTSD symptom severity and deployment-related mild TBI would report increased behavioral dyscontrol. Our results partially supported this hypothesis, demonstrating that PTSD symptom severity and deployment-related mild TBI were associated with behavioral dyscontrol in univariable and multivariable models adjusting for race, ethnicity, and sex. Further, results indicated a significant interaction effect between deployment-related mild TBI and PTSD symptoms, in which the effect of deployment-related mild TBI was significant only among those with *lower* severity of PTSD symptoms (PCL-5 scores of 0–23). This finding suggests a clear effect of deployment-related mild TBI when PTSD symptoms are lower, but that when PTSD symptoms are more severe, PTSD symptoms likely account for effects on behavioral dyscontrol. Moreover, those with high social support and self-efficacy scores also reported significantly lower scores on behavioral dyscontrol.

The observed elevation in dyscontrol scores among SM/Vs with both blast and non-blast deployment-related mild TBI, as opposed to those with blast-only TBI, provides a noteworthy point of discussion in the broader context of TBI research. The documented difference in dyscontrol scores, even after adjusting for other relevant factors, underscores the complexity of deployment-related mild TBI-related outcomes and the importance of considering injury mechanisms. The distinct neurological effects associated with blast and non-blast injuries may contribute uniquely to dyscontrol, reflecting the complex interplay between injury characteristics and resulting behavioral sequelae. Moreover, the absence of PTSD symptoms in the studied population emphasizes the specific impact of TBI subtypes on dyscontrol, independent of comorbid psychological conditions.

The association between elevated social support scores and diminished behavioral dyscontrol scores among SM/Vs emphasizes the potential importance of social support as a protective factor. Existing research demonstrates that social support from the military unit, friends, and family buffer the relationship between stressor exposure and posttraumatic stress symptoms. Social support from familial, peer, or community relationships is a stabilizing force that helps SM/V navigate the complexities of life after military service (26). Support networks can offer emotional reassurance, facilitate coping strategies, and provide a sense of belonging and understanding (26). Wilks and colleagues analyzed 2,467 Iraq/Afghanistan-era Veterans and found that TBI was associated with suicidal ideation, and social support was negatively associated with suicide ideation. Conversely, limited social support has been linked to heightened levels of stress, increased symptom severity, and a more challenging rehabilitation journey (27, 28). These findings, coupled with the findings in our study, help explain the connections between PTSD symptom severity and behavioral dyscontrol symptoms, and may bolster the need for additional social and family support as treatment adjuncts to lower the risk of these adverse outcomes.

Similarly, our results suggest that greater self-efficacy may also be protective and lead to decreased behavioral dyscontrol. The ability to effectively navigate challenges impacts cognitive, behavioral, affective, and functional outcomes and demonstrates a significant protective influence (29, 30). Research consistently demonstrates that high levels of self-efficacy increase resiliency among SM/Vs and are closely associated with better mental health outcomes (29, 30). Self-efficacy may also be an important factor in treatment. In one study of SMs receiving cognitive rehabilitation treatment (29), perceived self-efficacy at the beginning of treatment was associated with treatment engagement, suggesting self-efficacy mediates treatment outcomes. Increasing patients’ level of self-efficacy may be important for successful treatment of psychological distress in SM/Vs (30). Greater self-efficacy is associated with better mental health outcomes, such as enhanced coping skills, increased resilience, and reduced levels of anxiety and depression (29). The perception of being capable of resolving problems and attaining personal goals may empower SM/Vs to exert greater control over their actions and reactions, subsequently mitigating symptoms of behavioral dyscontrol (31).

### Study strengths

The present analysis has several inherent strengths. The sample size was substantial, representing 1,808 well-characterized SM/Vs with combat exposure from the LIMBIC-CENC multi-center cohort. This ensured robustness as we rigorously evaluated their lifetime mild TBI histories and benefited from the extensive data collected in the comprehensive assessments. The study controlled for symptom validity by excluding participants with non-credible symptom profiles on a validated measure.

### Study limitations

While our study sheds light on the effects of PTSD and deployment-related mild TBI on behavioral dyscontrol, it is essential to acknowledge several limitations. A significant limitation is that many of the study measures were self-reported and therefore cannot provide definitive clinical diagnoses. Additionally, inherent to the cross-sectional design, causal inferences and temporal dynamics remain constrained. Notably, the study lacks a systematic assessment of structural brain abnormalities, medications, and psychological evaluations at baseline which may have explained variance in our results.

### Implications for future research

Further research is warranted to identify how self-efficacy and social support influence behavioral dyscontrol in SM/Vs with PTSD and/or deployment-related mild TBI. Longitudinal studies can contribute to understanding the temporal relationship between self-efficacy and behavioral dyscontrol symptoms, elucidating whether changes in self-efficacy precede or follow improvements in behavioral regulation. Investigating the influence of social support can provide valuable insights into the interpersonal factors that contribute to behavioral dyscontrol symptoms and may inform interventions to improve behavioral regulation. Examining the types of support (e.g., emotional, instrumental, informational) and the sources of support (e.g., family, friends, healthcare providers) can provide a comprehensive understanding of how different aspects of social support impact behavioral regulation (32). A thorough exploration is critical to understanding the complex relationship of deployment-related mild TBI, PTSD symptom severity, and related psychosocial constructs in shaping behavioral dyscontrol among SM/Vs. Future research using longitudinal designs should provide a more comprehensive understanding of the temporal dynamics between deployment-related mild TBI, PTSD, and dyscontrol, which may influence the observed associations between these conditions.

## Conclusion

PTSD and mild TBI are commonly diagnosed during or following military deployments, and both are associated with behavioral dyscontrol in SM/Vs. The present analysis demonstrated that PTSD symptom severity and deployment-related mild TBI were each associated with behavioral dyscontrol in univariable and multivariable models adjusting for race, ethnicity, and sex. Deployment-related mild TBI primarily contributes to behavioral dyscontrol in the absence of prominent PTSD symptom severity. The findings highlight the complex relationship between PTSD symptoms and mild TBI resulting from deployment, particularly with regard to behavioral dyscontrol. Consequently, there is a pressing need for a comprehensive understanding and targeted interventions within clinical, research, and policy spheres, given the interdependence of these conditions.

## Data availability statement

The datasets presented in this study can be found in online repositories. The names of the repository/repositories and accession number(s) can be found at: Federal Interagency Traumatic Brain Injury Research (FITBIR) Informatics System. https://fitbir.nih.gov/.

## Ethics statement

The studies involving humans were approved by the local Institutional Review Boards at all eleven prospective longitudinal study enrollment sites. The studies were conducted in accordance with the local legislation and institutional requirements. Written informed consent for participation was not required from the participants or the participants’ legal guardians/next of kin in accordance with the national legislation and institutional requirements.

## Author contributions

KS: Conceptualization, Data curation, Formal analysis, Investigation, Methodology, Project administration, Resources, Visualization, Writing – original draft, Writing – review & editing. SMa: Formal analysis, Methodology, Visualization, Writing – review & editing. WW: Conceptualization, Data curation, Formal analysis, Funding acquisition, Methodology, Writing – review & editing. ZO: Conceptualization, Data curation, Methodology, Software, Validation, Visualization, Writing – original draft, Writing – review & editing. TP: Methodology, Writing – review & editing. SMi: Methodology, Writing – review & editing. CD-G: Writing – review & editing. KC: Writing – review & editing, Methodology. JR: Formal analysis, Methodology, Writing – review & editing. MO’N: Writing – review & editing, Investigation, Methodology. MP: Conceptualization, Investigation, Methodology, Resources, Supervision, Writing – review & editing.

## References

[1] Arciniegas DB Wortzel HS. Emotional and behavioral dyscontrol after traumatic brain injury. Psychiatr Clin North Am. (2014) 37:31–53. doi: 10.1016/j.psc.2013.12.001, PMID: 24529422

[2] Mac Donald CL Johnson AM Wierzechowski L Kassner E Stewart T Nelson EC. Outcome trends after US military concussive traumatic brain injury. J Neurotrauma. (2017) 34:2206–19. doi: 10.1089/neu.2016.4434, PMID: 27198861 PMC5510713

[3] Haarbauer-Krupa J Pugh MJ Prager EM Harmon N Wolfe J Yaffe K. Epidemiology of chronic effects of traumatic brain injury. J Neurotrauma. (2021) 38:3235–47. doi: 10.1089/neu.2021.0062, PMID: 33947273 PMC9122127

[4] Sayer NA Carlson KF Frazier PA. Reintegration challenges in U.S. service members and veterans following deployment-related TBI. Soc Issues Policy Rev. (2014) 8:33–73. doi: 10.1111/sipr.12001

[5] Elbogen EB Johnson SC Wagner HR Sullivan C Taft CT Beckham JC. Violent behaviour and post-traumatic stress disorder in US Iraq and Afghanistan veterans. Br J Psychiatry. (2014) 204:368–75. doi: 10.1192/bjp.bp.113.134627, PMID: 24578444 PMC4006087

[6] Burke H Degeneffe C Olney M. A new disability for rehabilitation counselors: Iraq war veterans with traumatic brain injury and post-traumatic stress disorder. J Rehabil. (2009) 75:5–14.

[7] Sayer NA Rettmann NA Carlson KF Bernardy N Sigford BJ Hamblen JL. Veterans with history of mild traumatic brain injury and post-traumatic stress disorder: challenges from provider perspective. J Rehabil Res Dev. (2009) 46:703–16. doi: 10.1682/JRRD.2009.01.0008, PMID: 20104400

[8] Rawat BPS Reisman J Pogoda TK Liu W Rongali S Aseltine RH Jr. Intentional self-harm among US veterans with traumatic brain injury or post-traumatic stress disorder: retrospective cohort study from 2008 to 2017. JMIR Public Health Surveill. (2023) 9:e42803. doi: 10.2196/42803, PMID: 37486751 PMC10407646

[9] Walker WC Carne W Franke LM Nolen T Dikmen SD Cifu DX. The chronic effects of neurotrauma consortium (CENC) multi-center observational study: description of study and characteristics of early participants. Brain Inj. (2016) 30:1469–80. doi: 10.1080/02699052.2016.1219061, PMID: 27834538

[10] Lippa SM Axelrod BN Lange RT. The mild brain injury atypical symptoms (mBIAS) scale in a mixed clinical sample. J Clin Exp Neuropsychol. (2016) 38:721–9. doi: 10.1080/13803395.2016.1161732, PMID: 27159359

[11] Tulsky DS Kisala PA Victorson D Carlozzi N Bushnik T Sherer M. TBI-QOL: development and calibration of item banks to measure patient reported outcomes following traumatic brain injury. J Head Trauma Rehabil. (2016) 31:40–51. doi: 10.1097/HTR.0000000000000131, PMID: 25931184 PMC4697960

[12] Walker WC Cifu DX Hudak AM Goldberg G Kunz RD Sima AP. Structured interview for mild traumatic brain injury after military blast: inter-rater agreement and development of diagnostic algorithm. J Neurotrauma. (2015) 32:464–73. doi: 10.1089/neu.2014.3433, PMID: 25264909

[13] Cooper DB Nelson L Armistead-Jehle P Bowles AO. Utility of the mild brain injury atypical symptoms scale as a screening measure for symptom over-reporting in operation enduring freedom/operation iraqi freedom service members with post-concussive complaints. Arch Clin Neuropsychol. (2011) 26:718–27. doi: 10.1093/arclin/acr070, PMID: 21873326

[14] Committee AMTBI. Definition of mild traumatic brain injury. J Head Trauma Rehabilitation. (1993) 8:86–7. doi: 10.1097/00001199-199309000-00010

[15] Management of Concussion/Mild TBI Working Group. VA/DoD clinical practice guideline for management of concussion/mild traumatic brain injury. J Rehabil Res Dev. (2009) 46:CP1–CP68. PMID: 20108447

[16] Blevins CA Weathers FW Davis MT Witte TK Domino JL. The post-traumatic stress disorder checklist for DSM-5 (PCL-5): development and initial psychometric evaluation. J Trauma Stress. (2015) 28:489–98. doi: 10.1002/jts.22059, PMID: 26606250

[17] James LM Strom TQ Leskela J. Risk-taking behaviors and impulsivity among veterans with and without PTSD and mild TBI. Mil Med. (2014) 179:357–63. doi: 10.7205/MILMED-D-13-00241, PMID: 24690958

[18] Bryant RA. Post-traumatic stress disorder and mild brain injury: controversies, causes and consequences. J Clin Exp Neuropsychol. (2001) 23:718–28. doi: 10.1076/jcen.23.6.718.1024, PMID: 11910539

[19] Herdman M Gudex C Lloyd A Janssen M Kind P Parkin D. Development and preliminary testing of the new five-level version of EQ-5D (EQ-5D-5L). Qual Life Res. (2011) 20:1727–36. doi: 10.1007/s11136-011-9903-x, PMID: 21479777 PMC3220807

[20] Schwarzer R Jerusalem M Weinman J Wright S Johnston M. Generalized self-efficacy scale In: Weinman J Wright S Johnston M, editors. Measures in health psychology: a user’s portfolio causal and control beliefs. Windsor: NFER-NELSON (1995)

[21] Vogt D Smith BN King LA King DW Knight J Vasterling JJ. Deployment risk and resilience inventory-2 (DRRI-2): an updated tool for assessing psychosocial risk and resilience factors among service members and veterans. J Trauma Stress. (2013) 26:710–7. doi: 10.1002/jts.21868, PMID: 24490250

[22] James G Witten D Hastie T Tibshirani R. An introduction to statistical learning, vol. 103. New York: Springer (2013).

[23] R Core Team. R: A language and environment for statistical computing. R Foundation for Statistical Computing. (2022) Vienna, Austria. Available at: https://www.R-project.org/

[24] Martindale SL Ord AS Lad SS Miskey HM Taber KH Rowland JA. Differential effects of deployment and non-deployment mild TBI on neuropsychological outcomes. Rehabil Psychol. (2021) 66:128–38. doi: 10.1037/rep0000374, PMID: 33382338 PMC8396071

[25] Rowland JA Stapleton-Kotloski JR Rogers E Taber KH Godwin DW Martindale SL. Contextual effects of traumatic brain injury on the connectome: differential effects of deployment- and non-deployment-acquired injuries. J Head Trauma Rehabil. (2022) 37:E449–57. doi: 10.1097/HTR.0000000000000803, PMID: 35862901 PMC9643591

[26] Elbogen EB Dennis PA Van Voorhees EE Blakey SM Johnson JL Johnson SC. Cognitive rehabilitation with Mobile technology and social support for veterans with TBI and PTSD: a randomized clinical trial. J Head Trauma Rehabil. (2019) 34:1–10. doi: 10.1097/HTR.0000000000000435, PMID: 30169439 PMC6314886

[27] Smith BN Vaughn RA Vogt D King DW King LA Shipherd JC. Main and interactive effects of social support in predicting mental health symptoms in men and women following military stressor exposure. Anxiety Stress Coping. (2013) 26:52–69. doi: 10.1080/10615806.2011.634001, PMID: 22098413

[28] Wilks CR Morland LA Dillon KH Mackintosh MA Blakey SM Wagner HR. Anger, social support, and suicide risk in U.S. military veterans. J Psychiatr Res. (2019) 109:139–44. doi: 10.1016/j.jpsychires.2018.11.026, PMID: 30537566

[29] Blackburn L Owens GP. The effect of self-efficacy and meaning in life on post-traumatic stress disorder and depression severity among veterans. J Clin Psychol. (2015) 71:219–28. doi: 10.1002/jclp.22133, PMID: 25270887

[30] Belanger HG Vanderploeg RD Curtiss G Armistead-Jehle P Kennedy JE Tate DF. Self-efficacy predicts response to cognitive rehabilitation in military service members with post-concussive symptoms. Neuropsychol Rehabil. (2020) 30:1190–203. doi: 10.1080/09602011.2019.1575245, PMID: 30764711

[31] Elder GA Ehrlich ME Gandy S. Relationship of traumatic brain injury to chronic mental health problems and dementia in military veterans. Neurosci Lett. (2019) 707:134294. doi: 10.1016/j.neulet.2019.134294, PMID: 31141716 PMC12199262

[32] Moore TM Risbrough VB Baker DG Larson GE Glenn DE Nievergelt CM. Effects of military service and deployment on clinical symptomatology: the role of trauma exposure and social support. J Psychiatr Res. (2017) 95:121–8. doi: 10.1016/j.jpsychires.2017.08.013, PMID: 28843074 PMC5653464

